# Gold nanoparticles alleviates the lipopolysaccharide-induced intestinal epithelial barrier dysfunction

**DOI:** 10.1080/21655979.2021.1972782

**Published:** 2021-09-15

**Authors:** Zhen Wang, Yinya Cao, Kangzhen Zhang, Zhirui Guo, Ying Liu, Ping Zhou, Zhengxia Liu, Xiang Lu

**Affiliations:** a Lab Center, The Second Affiliated Hospital of Nanjing Medical University, Nanjing, China; b Department of Critical Care Medicine, Yijishan Hospital, First Affiliated Hospital of Wannan Medical College, Wuhu, China; c Department of Geriatrics, Sir Run Run Hospital, Nanjing Medical University, Nanjing, China

**Keywords:** Gold nanoparticles, LPS, intestinal inflammation

## Abstract

Nanotechnology is used in the immune response manipulation to treat various human diseases. In the present study, we explored the effects of Au nanoparticles (AuNPs) on the lipopolysaccharide (LPS)-induced epithelial barrier dysfunction and inflammatory response of colonic epithelial NCM460 cells. According to the results of cell counting kit-8 and flow cytometry analysis, the viability of NCM460 cells was inhibited, and the apoptosis was increased after LPS treatment, and AuNPs reversed these changes in a dose-dependent way. The permeability was evaluated by detecting the flux of fluorescein isothiocyanate-dextran and transepithelial electrical resistance. LPS enhanced the permeability and promoted barrier dysfunction of NCM460 cells. Enzyme-linked immunosorbent sorbent assay results revealed that the concentrations of pro-inflammatory factors and nitric oxide were elevated by LPS treatment and decreased by the AuNPs. LPS aggravated the inflammatory response, which was rescued by the AuNPs. Moreover, LPS promoted the activation of the nuclear factor kappa-B and extracellular signal-regulated kinase/c-Jun NH-terminal kinase signaling pathways, which were inhibited by AuNPs.

## Introduction

Gastrointestinal dysfunction is an essential risk factor for patients with sepsis, in the context of the high mortality rate of sepsis and septic shock [1,2]. Corrupted intestine barrier function plays the dual roles of ‘victim’ and ‘perpetrator’ during sepsis [3,4]. Inflammatory cytokines induced by sepsis, including interleukin-1 beta (IL-1β) and tumor necrosis factor α (TNF-α), affect epithelial tight junction proteins, following hyperpermeability of intestinal lumen and translocation of potentially pathogenic microbes into lymph nodes or the systemic circulation [5–7]. The tight junction (TJ), comprising of transmembrane proteins such as occludin and claudins, is a multiprotein complex at the apical end of the lateral membrane and selectively modulates paracellular transport as well as maintains the polarity of cells [8,9]. The defection of intestinal epithelial TJs is revealed to be a pathogenic marker of inflammatory bowel disease [10]. Increasing evidence indicates that the treatment of LPS damages the structure of TJs and induces elevated TJ permeability [11,12].

Nanotechnology provides efficient methods in the therapy of various diseases. Nanodevices are usually designed for specific biomedical applications in hip joint replacement [13], vaccine development [14], prevention of transplant rejection [15], and treatment of inflammatory diseases by enhancing or inhibiting the immune responses [16]. Increasing attention has been paid to the gold nanoparticles (AuNPs) due to their unique plasmonic characteristics and extensive application [17,18]. The AuNPs-modified screen-printed electrodes were used to convert and amplify the electrochemical current signals upon presence of levodopa molecules, which can benefit treatment of Parkinson’s disease [19]. A smartphone-based integrated voltammetry system with AuNPs-modified screen-printed electrodes showed great potentials in point-of-care testing [20]. AuNPs, as anti-inflammatory agents, have drawn increasing interest in the regulation of inflammatory responses in specific tissues or organs [21]. AuNPs were confirmed to alleviate lipopolysaccharide (LPS)-induced inflammatory response via inactivating the nuclear factor kappa-B (NF-κB) and Janus kinase 2 (JAK2)/signal transducer and activator of transcription 3 (STAT3) signaling pathways in RAW 264.7 macrophages [22]. Additionally, AuNPs could reduce high glucose-induced inflammation in macrophages via the tuberin-mammalian target of rapamycin (mTOR)/NF-κB pathway [23]. However, the application of AuNPs in the treatment of intestinal inflammation remains not fully understood. Moreover, owing to high cytotoxicity from the usage of cationic surfactant cetyltrimethyl ammonium bromide (CTAB) in synthetic process, the potential biomedical application of AuNPs fell into the dilemma [24]. We have developed a unique stepwise surfactant exchange process that converts CTAB-stabilized AuNPs into citrate-stabilized AuNPs, which retains impressive physiochemical characteristic and lower cytotoxicity [25].

We hypothesized that AuNPs play a role in LPS-induced colonic epithelial injury. The aim of our study was to explore and analyze the effect and mechanisms of AuNPs against LPS-induced responses of colonic epithelial NCM460 cells, which may expand our knowledge of the application of AuNPs in the treatment of intestinal inflammation.

## Material and methods

### Cell culture

The human normal colonic epithelial cell line (NCM460; EK-Bioscience, Shanghai, China) was cultured in Dulbecco’s Modified Eagle Medium (DMEM, Thermo Fisher, Shanghai, China) with 10% fetal bovine serum (FBS; Thermo Fisher) and 1% penicillin–streptomycin mixed solution at 37°C in 5% CO_2_. For LPS (Thermo Fisher) treatment, 1 μg/mL of LPS was added into the basolateral compartments for 24 h.

### Preparation and characterization of citrate-stabilized auNPs

The citrate-stabilized AuNPs were prepared based on the previous studies [25,26]. A seed-mediated method was used to synthesize CTAB-stabilized AuNPs in an aspect ratio of 4:1. Subsequently, 100 mL of binary surfactant solution of CTAB (0.037 M) and NaOL (0.078 M) with 1.44 mL of AgNO_3_ (10 mM) and 2 mL of HAuCl_4_ (25 mM) were added into the growth solution at 30°C for 30 minutes and stirred. Next, the growth solution was added with seed suspension (0.08 mL) and maintained overnight at 30°C. NaBH_4_ (0.6 mL; 10 mM) was added into 10 mL of HAuCl_4_ (0.25 mM) in 0.1 M CTAB solution and vigorously stirred for 3 min to prepare CTAB-stabilized Au seeds. The Au seeds were successfully formed when the solution color changed from yellow to brown. BH-4 experienced complete hydrolysis in the seed suspension was maintained at room temperature for over 2 h.

The CTAB in AuNPs suspension was removed after centrifugation (9500 × *g*) for 15 min. The suspension was diluted with water to a level when its optical density was the same as original AuNPs (3.0). Original AuNPs (80 mL) was supplemented into 8 mL of PSS (10 g/L in 5 mM NaCl) aqueous solution and vigorously stirred and then maintained for 1 h. Next, the mixture was centrifuged (9000 g) for 15 min and dispersed with water (80 mL) repeatedly two times. Dispersion was performed using 20 mL of water and 60 mL of citrate (5 mM) and kept for 12 h, and the AuNPs suspension was centrifugated (9000 g) for 20 min and added with 10 mL of water and 30 mL of citrate solution (5 mM).

### Detection of cell viability

The viability of NCM460 cells was assessed using a cell counting kit 8 (CCK-8, Dojindo, Tokyo, Japan). NCM460 cells (100 μL) were cultured in 96-well plates and incubated for 24 h treated with different concentrations of LPS (0, 5, 10, 15, 20, 25 μg/ml). The CCK-8 solution (10 μL) was supplemented into each well and incubated for another 4 h at room temperature. The absorbance at 450 nm was measured with a microplate reader (Reagen, Shenzhen, China).

### Flow cytometry analysis

The apoptosis of NCM460 cells was assessed using annexin V-fluorescein isothiocyanate (FITC) and propidium iodide (PI) staining. Phosphate-buffered saline (PBS) was used to wash the NCM460 cells, which were then resuspended in the binding buffer. Subsequently, annexin V-FITC (5 μl, 50 μg/mL) and PI (5 μl, 50 μg/mL) were used to counterstain the NCM460 cells for 15 minutes in the darkness. The apoptosis was detected using a flow cytometer (BD Biosciences, Franklin Lakes, NJ, USA).

### Quantitative real-time polymerase chain reaction (qRT-PCR)

Total RNA in NCM460 cells was purified using the TRIzol reagent. Total RNAs (500 ng) were reverse transcribed into cDNAs with a RevertAid First-Strand cDNA Synthesis Kit (Thermo Fisher, Waltham, MA, USA). PCR was performed with a SYBR Green I Master kit (Roche, Germany). The mRNA expression of inducible nitric oxide synthase (iNOS) was calculated using the 2^−∆∆Ct^ method [27] with glyceraldehyde 3-phosphate dehydrogenase (GAPDH) as an internal control. The primer sequences are as follows:

iNOS:

F: 5ʹ-ACGTGCACCAAACTTTAGG-3ʹ,

R: 5ʹ-CAAACGGAGCTAATCTCCA-3ʹ;

GAPDH:

F: 5ʹ-TCATTTCCTGGTATGACAACGA-3ʹ,

R: 5ʹ-GGTCTTACTCCTTGGAGGC-3ʹ;

### Western blot analysis

Total proteins were extracted from the NCM460 cells using radioimmunoprecipitation assay lysis buffer. The lysates of NCM460 cells were centrifuged at 140,000 × *g* for 30 min followed by supernatant collection. Then the proteins were isolated with the sodium dodecyl sulfate polyacrylamide gel electrophoresis gel and transferred onto the polyvinylidene fluoride membranes (Millipore, Billerica, MA, USA). Subsequently, the membranes were blocked with 5% nonfat milk and cultured with the primary antibodies overnight at 4°C, including anti-B-cell lymphoma 2 associated X (Bax; #ab182734, 1/1000; Abcam), anti-B-cell lymphoma 2 (Bcl-2; #ab59348, 1/500; Abcam), anti-cleaved caspase-3 (C-caspase-3; #ab2302, 1/500; Abcam), anti-zonula occludens 1 (ZO-1; #ab276131, 1/1000; Abcam), anti-occludin (#ab216327, 1/1000; Abcam), anti-iNOS (#ab178945, 1/1000; Abcam), anti-cyclooxygenase-2 (COX2; #ab62331, 1/1000; Abcam), anti-phosphorated IκB kinase alpha/beta (p-IKKα/β; #2697, 1/1000; Cell signaling), anti-p-p65 (#ab76302, 1/1000; Abcam), anti-p65 (#ab76311, 1/1000; Abcam), anti-phosphorylated extracellular signal-regulated kinase (p-ERK; #ab201015, 1/1000; Abcam), anti-ERK (#ab32537, 1/1000; Abcam), anti-phosphorated c-Jun NH-terminal kinase (p-JNK; #ab47337, 1/1000; Abcam), anti-JNK (#ab199380, 1/2500; Abcam), anti-p-p38 (#ab178867, 1/1000; Abcam), anti-p38 (#ab170099, 1/1000; Abcam). After being washed with PBS, the membranes were cultured with secondary antibody (#ab150073, 1/500; Abcam) for 1 h in the darkness at room temperature. The protein bands were visualized using enhanced chemiluminescence solution purchased from Thermo Fisher.

### Enzyme-linked immunosorbent assay (ELISA)

NCM460 cells were centrifuged at 5,000 × *g* at 4°C for 10 min, and then the supernatant was obtained. The concentrations of IL-6, TNF-α and NO in cell supernatant were detected using an IL-6 ELISA kit (RAB0306; Sigma-Aldrich), a TNF-α ELISA kit (ab181421; Abcam), and a nitric oxide ELISA kit (1227168631; klamar). The optical density value at 450 nm was assessed at the end of the experiment using a microplate reader.

### Measurement of transepithelial electrical resistance (TEER)

Caco2 cells (1 × 10^5^) were cultured in 0.33 cm^2^ polyethylene terephthalate membrane inserts with 0.4 mm pores. The permeability of the NCM460 cells was assessed by the measurement of transepithelial electrical resistance using the Millicell-ERS-2 electrical resistance system (Sigma-Aldrich, Shanghai, China). The NCM460 cells were washed with PBS. The TEER value was measured consecutively in triplicates. The TEER values were shown as ohm cm^2^.

### Paracellular permeability assay

The epithelial permeability of NCM460 cells was assessed by detecting the flux of fluorescein isothiocyanate (FITC)-dextran (Sigma-Aldrich, Shanghai, China). The NCM460 cell medium was removed, and the apical side of the inserts was added with the FITC-dextran (1 mg/mL), while the basolateral side was supplemented with serum-free growth medium (1.5 mL). After culturing for 6 h, the basolateral medium (100 μL) was obtained, and a microplate reader was used to detect the fluorescence at 480 nm excitation wavelength and 520 nm emission wavelength.

### Statistical analysis

Each biological sample was run in triplicate and experiments were independently repeated thrice. The value of data was expressed as the mean ± standard deviation. The statistical analyses were conducted using SPSS 17.0 (IBM, Armonk, NY, USA) with Student’s *t*-test for evaluation of difference between two groups, and one-way analysis of variance for difference evaluation among multiple groups. *p*-Value <0.05 was regarded as statistically significant.

## Results

### LPS inhibits the growth and promotes the apoptosis of NCM460 cells

**Figure 1.**
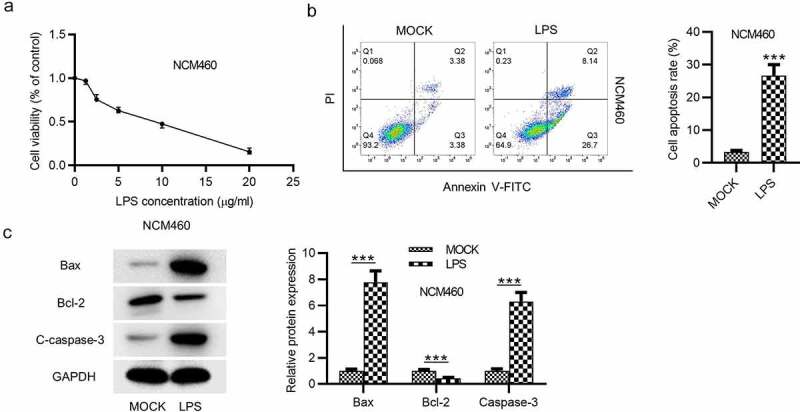
LPS induces the apoptosis of NCM460 cells. (a) A CCK-8 assay was conducted to access the NCM460 cell viability after being treated with increasing levels of LPS (0, 5, 10, 15, 20 μg/ml). (b) The apoptosis of NCM460 cells was examined by flow cytometry analysis. (c) The expression of apoptosis-associated proteins (Bax, Bcl-2, C-caspase-3) in LPS-stimulated NCM460 cells was detected by western blot. ***p < 0.001

First, the effects of LPS on the viability and apoptosis of NCM460 cells were detected. The viability of NCM460 cells treated with increasing concentration of LPS was measure by CCK-8 assay, and the result showed that the viability of NCM460 cells was suppressed by LPS in a dose-dependent way (Figure 1(a)). The result of flow cytometry analysis indicated that LPS treatment promoted the apoptosis of NCM460 cells (Figure 1(b)). Western blot was performed to examine the levels of apoptosis-associated proteins. The levels of C-caspase-3 and Bax were elevated after LPS treatment, while the level of Bcl-2 was reduced in LPS-stimulated NCM460 cells (Figure 1(c)).

### LPS leads to NCM460 cell epithelial barrier dysfunction

**Figure 2.**
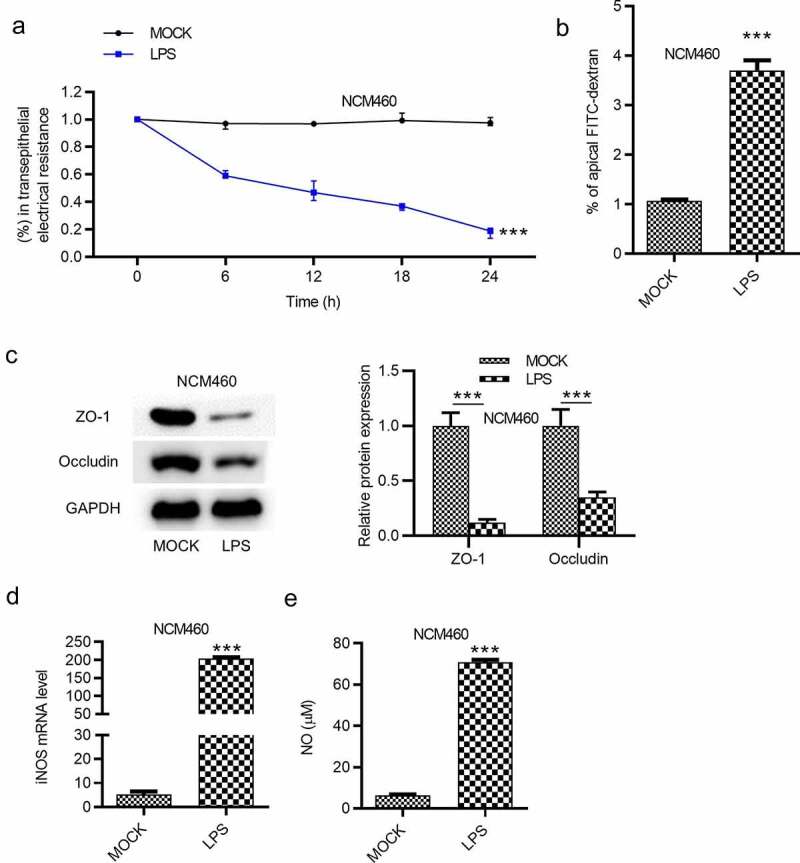
LPS induces epithelial barrier dysfunction of NCM460 cells. (a) Transepithelial electrical resistance (TEER) of NCM460 cells after LPS treatment was detected to analyze the barrier function. (b) Non-absorbable fluorescein isothiocyanate (FITC)-conjugated dextran was used to access the permeability of NCM460 cells. (c) The expression of tight junction proteins (ZO-1, occludin) in LPS-stimulated NCM460 cells was detected by western blot. (d and e) The RT-qPCR and ELISA were used to measure the mRNA expression of iNOS and the concentration of NO in LPS-treated NCM460 cells. ***p < 0.001

Effects of LPS on epithelial barrier dysfunction of NCM460 cells were subsequently detected. Transepithelial permeability was assessed by the value of transepithelial electrical resistance (TEER) and the flux of the paracellular solute FITC-dextran in NCM460 cells. The result demonstrated that the treatment of LPS reduced the value of TEER in NCM460 cells (Figure 2(a)). FITC-dextran permeability was significantly elevated by LPS treatment in NCM460 cells, as shown in (Figure 2(b)). Western blot analysis was also used to measure the levels of tight junction proteins. The result showed that ZO-1 and occludin expression was decreased in NCM460 cells stimulated with LPS (Figure 2(c)). Previous studies have demonstrated that iNOS and NO are implicated in the LPS-induced barrier dysfunction [28,29]. We measured the iNOS mRNA expression and the NO concentration in NCM460 cells. The results showed that iNOS mRNA and NO concentration were both significantly elevated by the LPS stimulation (Figure 2(d-e)).

### LPS induces the inflammatory response

**Figure 3.**
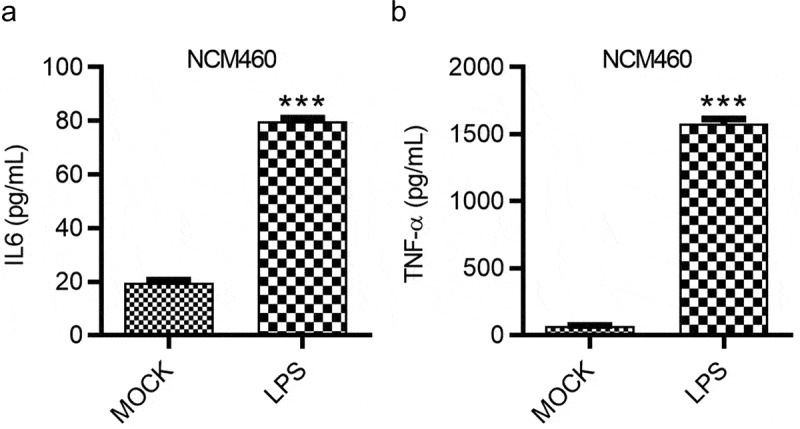
LPS induces the inflammatory response of NCM460 cells. (a and b) The concentrations of IL-6 and TNF-α in NCM460 cells after indicated treatment were measured using ELISA. ***p < 0.001

Influences of LPS on the inflammatory response of NCM460 cells were evaluated by ELISA. The concentration of pro-inflammatory factors IL-6 and TNF-α in NCM460 cells showed a significant increase after LPS treatment. The increase of IL-6 concentration was more than threefold while that of TNF-α was more than 20-fold (Figure 3(a-b)).

### AuNPs ameliorate the LPS-induced NCM460 cell injury

The rescue effects of AuNPs on LPS-induced damage of NCM460 cells were explored. The LPS-induced increase in the concentrations of pro-inflammatory factors IL-6 and TNF-α in NCM460 cells was reversed by the introduction of AuNPs in a dose-dependent way (Figure 4(a-b)). Moreover, LPS stimulation caused elevated mRNA expression of iNOS and increased concentration of NO in NCM460 cells, which were rescued by the treatment of AuNPs. The rescue effect of AuNPs was concentration-dependently increased (Figure 4(c-d)). According to the CCK-8 assay, the treatment of AuNPs rescued the decrease in cell viability caused by LPS. Higher concentrations of AuNPs showed better rescue effect in NCM460 cells (Figure 4(e)). The cell apoptosis was examined using flow cytometry analysis. AuNPs reversed the LPS-stimulated increase in cell apoptosis in a dose-dependent way (figure 4(f-g)). The increase in protein expression of Bax and C-caspase-3 after LPS treatment was reversed by AuNPs. The Bcl-2 showed opposite expression change (Figure 4(h-i)). The levels of tight junction proteins (ZO-1, occludin) and proinflammatory factors (iNOS, COX2) were also detected using western blot. The result showed that the protein levels of iNOS, COX2 were increased by LPS treatment, which was reversed by AuNPs in a dose-dependent way. On the contrary, the ZO-1 and occludin expression was reduced after treatment of LPS, while the treatment of AuNPs reversed the LPS-mediated reduction in ZO-1 and occludin protein expression in a dose-dependent way (Figure 4(j-k)). The LPS-mediated decrease in the value of transepithelial electrical resistance (TEER) was reversed by the application of AuNPs in a dose-dependent way in NCM460 cells (Figure 4l). Furthermore, the increase in the flux of solute FITC-dextran caused by LPS was counteracted by the treatment of AuNPs in NCM460 cells (Figure 4(m)). The results indicated that AuNPs reversed the influence of LPS on the viability, apoptosis, inflammation and epithelial barrier function of NCM460 cells.**Figure 4.**
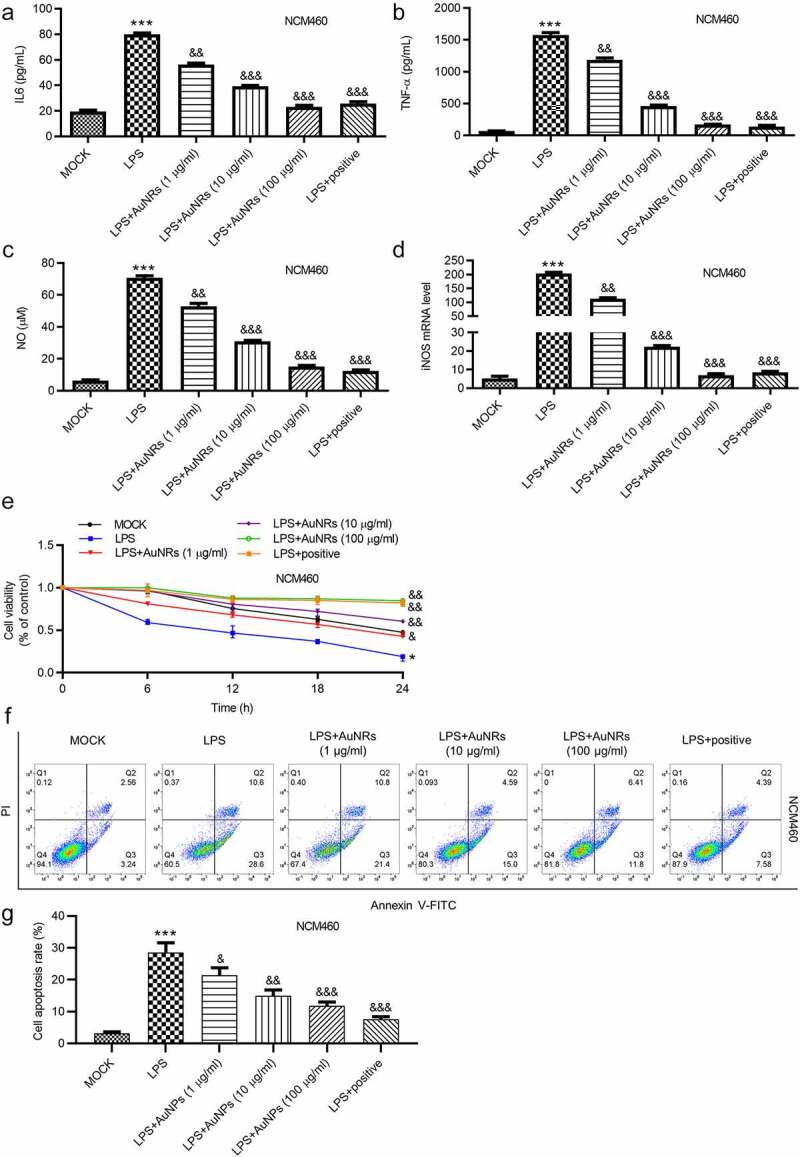
AuNPs ameliorate the LPS-induced NCM460 cell injury. (a–d) The concentrations of IL-6, TNF-α and NO and mRNA expression of iNOS in NCM460 cells treated with LPS or LPS in combination with elevated concentrations of AuNPs (1, 10, 100 μg/ml). (e) A CCK-8 assay was performed to examine the viability of NCM460 cells treated with LPS alone or in combination with different concentrations of AuNPs. (f and g) The apoptosis of NCM460 cells treated with LPS alone or in combination with different concentrations of AuNPs was examined by flow cytometry analysis. (h and i) The protein levels of Bax, Bcl-2, C-caspase-3 were analyzed by western blot. (j and k) The protein levels of tight junction proteins (occludin, ZO-1) and pro-inflammatory factors (iNOS, COX2) were detected by western blot. (l) The barrier function of NCM460 cells treated with LPS or LPS with AuNPs was examined by transepithelial electrical resistance (TEER) assay. (m) The permeability of NCM460 cells after treatment of LPS or LPS and AuNPs was assessed by paracellular solute FITC-dextran. *p < 0.05, ***p < 0.001, ^&^*p* < 0.05, ^&&^*p* < 0.01, ^&&&^*p* < 0.001

**Figure:**
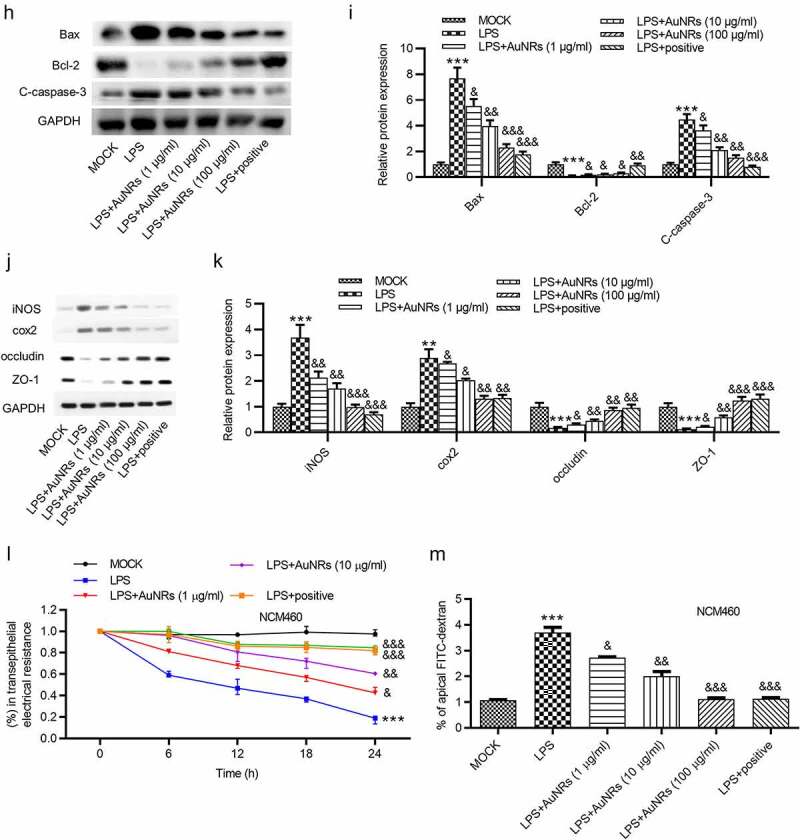
(countined)

### AuNPs inactivates the NF-κB and ERK/JNK signaling

**Figure 5.**
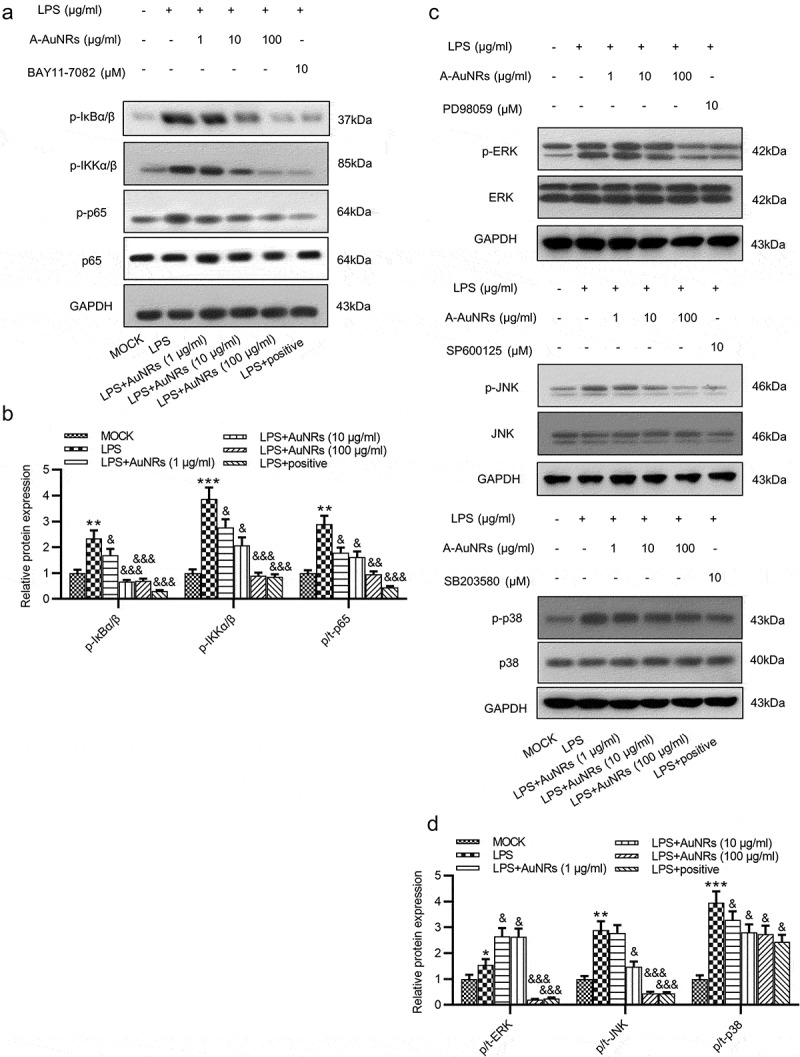
AuNPs inhibits the activation of the NF-κB and ERK/JNK signaling pathways. (a–d) Western blot was used to examine the levels of key proteins on the NF-κB and ERK/JNK signaling such as p-IκBα/β, p-IKKα/β, p-p65, p65, p-ERK, ERK, p-JNK, JNK, p-p38 and p38 after LPS treatment alone or in combination with AuNPs in NCM460 cells. BAY11-7082 was used as the inhibitor of NF-κB, while the PD98059 was used as inhibitor of ERK/JNK. *p < 0.05, **p < 0.01, ***p < 0.001, ^&^*p* < 0.05, ^&&^*p* < 0.01, ^&&&^*p* < 0.001

Whether the inflammatory signaling pathways are activated in NCM460 cells by LPS was further explored. The NF-κB and the ERK/JNK pathways were critically implicated in various diseases [30,31]. The levels of key proteins on the NF-κB (p-IκBα/β, p-IKKα/β, p-p65, p65) and ERK/JNK signaling (p-ERK, ERK, p-JNK, JNK, p-p38, p38) were examined using western blot, and the result showed that the protein levels of p-IκBα/β, p-IKKα/β, p-p65, p-ERK and p-JNK as well as the ratios of p-p65/p65, p-ERK/ERK and p-JNK/JNK, p-p38/p38 were all elevated after LPS treatment, while these effects were reversed by the introduction of AuNPs in a dose-dependent way in NCM460 cells (Figure 5(a-d)).

## Discussion

The mucosa of guts are comprised of epithelial cells, which serves as a significant barrier with defense functions. Numerous external microbes and environmental antigens enter the gut lumen mainly through the mucosa. Intestinal epithelium integrity is vital for preventing luminal infection, which is closely related to immune sensing [24]. The integrity or defects of the epithelial barrier are critical pathogenic markers related to inflammatory bowel disease [25]. The intestinal barrier dysfunction leads to increased epithelium permeability and causes an inflammatory response [26]. Previous studies have documented that LPS promotes the inflammatory response via increasing the production of pro-inflammatory cytokines and pathways. For example, LPS induces inflammatory response in macrophages and intestinal epithelial cells via facilitating the productions of NO and proinflammatory cytokines (IL-6, IL-1β, and TNF-α) [32]. In the present study, after LPS treatment, the concentrations of proinflammatory factors IL-6, TNF-α, iNOS, and COX2 in NCM460 cells were increased, which further verifies the effect of LPS on inducing inflammatory response by facilitating the expression of pro-inflammatory cytokines. Moreover, LPS triggered decreased cell viability and increased cell apoptosis, which was supported by the LPS-stimulated upregulation of BAX and C-caspase-3 as well as downregulation of Bcl-2. Importantly, levels of TJ proteins including ZO-1 and occludin were reduced by LPS treatment. The TEER of NCM460 cells was reduced while the FITC-dextran flux was increased by LPS treatment, indicating that LPS disrupted NCM460 cell permeability. All these data demonstrated that LPS effectively induced cell injury and dysfunction, which is consistent with previous studies [33–35].

Nanoparticles are suggested to be used as antimicrobial agents due to their high-performance in security and durability [36]. Particularly, AuNPs exerted important effects on inflammation and tight junction proteins, for example, AuNPs are reported to suppress the neuroinflammation in Alzheimer’s disease [19]. Furthermore, AuNPs modulate tight junctions in brain endothelial cells and colon cancer cells [37,38]. Likewise, in NCM460 cells, AuNPs reversed the effect of LPS treatment on tight junction proteins including ZO-1 and occludin and proinflammatory cytokines including IL-6, TNF-α, iNOS, and COX2. The LPS-stimulated decrease in TEER and increase in FITC-dextran flux were rescued by AuNPs, suggesting that AuNPs attenuated LPS-induced permeability. Moreover, AuNPs can upregulate Bcl-2 and downregulate BAX and C-caspase-3 to reverse the LPS-stimulated apoptosis of NCM460 cells. In conclusion, AuNPs improved LPS-induced cell injury and cell dysfunction.

Previous studies indicated that AuNPs alleviate inflammatory response and tight junction proteins via several signaling pathways including the NF-κB and ERK/JNK pathways [23,39–41]. In detail, AuNPs protected against Aβ-induced neuroinflammation and neurodegeneration by targeting the NF-κB/JNK/glycogen synthase kinase 3β (GSK3β) signaling pathway [39]. Hence, we hypothesized that AuNPs may function in a similar pattern in NCM460 cells. Our findings revealed that LPS induced the protein levels of p-IκBα/β and p-IKKα/β and ratios of p-p65/total p65, p-ERK/total ERK, p-JNK/JNK, p-p38/p38, while these effects were rescued by AuNPs, confirming that AuNPs inactivate the LPS-stimulated NF-κB and ERK/JNK pathways. Compared with a previous study which revealed that AuNPs alleviate LPS-induced inflammatory response by the NF-κB and JAK2/STAT3 signaling pathways in macrophages [22], the present study further validated that AuNPs rescued the LPS-induced inflammatory response by the NF-κB pathway in NCM460 cells, and meanwhile, we innovatively revealed that AuNPs can inactivate the ERK/JNK pathway and alleviate LPS-induced apoptosis and permeability of NCM460 cells.

However, there were also some limitations of the present study. We did not perform the animal experiments, and *in vivo* data that indicate AuNPs regulate the LPS-induced intestinal inflammation will be achieved in the future. We only verified the effect of AuNPs in NCM460 cells, and more explorations in other colonic epithelial cells are needed.

## Conclusion

This study revealed that LPS induced suppression in viability and paracellular tight junction of colonic epithelial NCM460 cells and stimulated increase in cell apoptosis, permeability, expression of inflammatory factors by the activation of the NF-κB and ERK/JNK pathways. AuNPs inactivated the NF-κB and ERK/JNK pathways to rescue the LPS-induced damage in NCM460 cells. This study may provide a theoretical basis for the treatment of intestinal inflammation using AuNPs.
